# Confirming the association of ulcerative colitis with interstitial lung disease: a retrospective analysis of comorbid disease, demographics, and healthcare utilization

**DOI:** 10.1093/crocol/otag085

**Published:** 2026-07-24

**Authors:** Sneh Parekh, Landen Shane Burstiner, Kaitlyn Romero, Anvit Reddy, Gerardo Diaz Garcia, Atefeh Vaezi, Mehdi Mirsaeidi, Lauren Stemboroski, Marianny Sulbaran

**Affiliations:** Department of Internal Medicine, University of Florida, College of Medicine – Jacksonville, Jacksonville, FL 32209, United States; Department of Internal Medicine, University of Florida, College of Medicine – Jacksonville, Jacksonville, FL 32209, United States; Division of Gastroenterology, University of Florida, College of Medicine – Gainesville, Gainesville, FL 32608, United States; Department of Internal Medicine, University of Florida, College of Medicine – Jacksonville, Jacksonville, FL 32209, United States; Department of Internal Medicine, University of Florida, College of Medicine – Jacksonville, Jacksonville, FL 32209, United States; Department of Internal Medicine, University of Florida, College of Medicine – Jacksonville, Jacksonville, FL 32209, United States; Division of Pulmonary, Critical Care, and Sleep Medicine, University of Florida, College of Medicine – Jacksonville, Jacksonville, FL 32209, United States; Division of Pulmonary, Critical Care, and Sleep Medicine, University of Florida, College of Medicine – Jacksonville, Jacksonville, FL 32209, United States; Division of Gastroenterology, University of Florida, College of Medicine – Jacksonville, Jacksonville, FL 32209, United States; Division of Gastroenterology, University of Florida, College of Medicine – Jacksonville, Jacksonville, FL 32209, United States

**Keywords:** inflammatory bowel disease, ulcerative colitis, Crohn’s disease, interstitial lung disease, extraintestinal manifestations

## Abstract

**Background:**

Interstitial lung disease (ILD) is a rare extraintestinal manifestation associated with inflammatory bowel disease (IBD). Limited information is available regarding the prevalence and risk factors of ILD in IBD. This study aimed to determine the association of ILD in Crohn’s disease (CD) and ulcerative colitis (UC), while analyzing various contributory factors.

**Methods:**

A retrospective analysis of patient data at two major tertiary healthcare centers in the southeastern United States from 2012 to 2022 was conducted. Patient demographics, healthcare utilization patterns, and coexisting diagnoses of IBD and ILD were analyzed using comparative statistical analysis.

**Results:**

A total of 8865 IBD patients were included in the study, of which 408 (4.6%) had concomitant IBD and ILD. Patients with UC had a significantly higher prevalence of ILD compared to those with CD (*P* < .001). There were no statistically significant differences in demographics between UC-ILD and CD-ILD patients. There was a significant difference in prevalence between White patients and minority patients with IBD and any form of ILD (*P* < .001). Finally, individuals with IBD-ILD were significantly older, had a higher proportion of emergency department (ED) visits, had a higher proportion of hospitalizations, had a higher proportion of patients with steroid use, and had a lower proportion of patients with mesalamine prescribed (*P* < .001), compared with IBD-only patients.

**Conclusions:**

Our findings confirm the established literature that ILD is more associated with UC compared to CD, while providing new insight into demographic and healthcare utilization factors between CD-ILD and UC-ILD patients and between IBD-ILD compared to IBD-only patients.

## Graphical Abstract

**Graphical Abstract:**
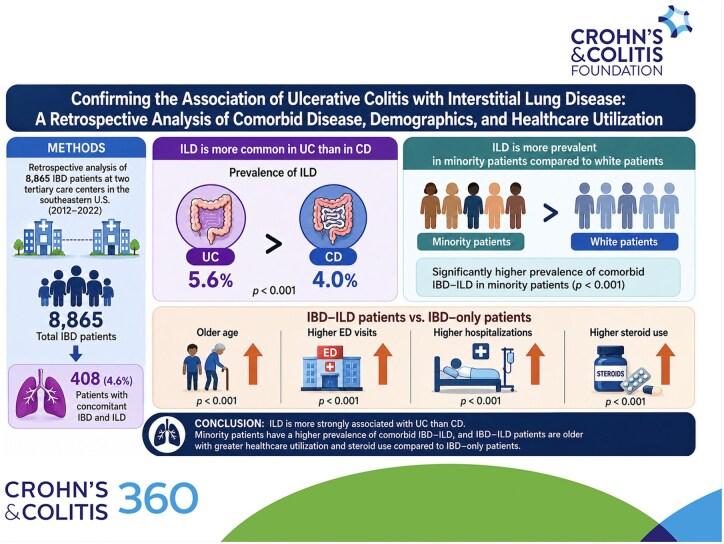

## Introduction

Inflammatory bowel disease (IBD) consists of both Crohn’s disease (CD) and ulcerative colitis (UC). Both diseases involve inflammation throughout the gastrointestinal (GI) tract, with variations in mechanism, location, and extraintestinal manifestations (EIMs). One such EIM associated with IBD is interstitial lung disease (ILD), which is rare and often underdiagnosed.^1^^,^^2^

EIMs of IBD are common in both CD and UC, and can involve any organ system including the musculoskeletal, dermatologic, renal, and pulmonary systems.^1^ While less commonly seen, pulmonary manifestations have been associated with IBD, with respiratory involvement in IBD reported in literature since 1976.^2^ Respiratory EIMs of IBD include chronic bronchitis, subglottic stenosis, bronchiectasis, bronchiolitis, and ILD.^1^^,^^2^

ILD is a group of pulmonary disorders characterized by inflammation and occasional fibrosis of the lung interstitium, which can result in impaired gas exchange, progressive dyspnea, and even respiratory failure.^3–5^ ILD has many different presentations, and diagnosing ILD can be challenging due to the need to rule out other respiratory disorders and the varying diagnostic criteria that exist depending on the etiology and subtype of ILD.

It has been proposed that pulmonary involvement in IBD results from the intricate interaction between the gut and lungs, known as the gut–lung axis. Alterations in the gut microbiome seen in IBD can result in activation of the mucosal immune system, inducing systemic immune activation, and elevating the inflammatory response in the lungs.^6^ One study by Metwali et al. explored this principle by exposing mice with IBD to bacterial endotoxin. Metwali et al. demonstrated that the endotoxin stimulated colonic inflammatory cells which then homed to the lungs, causing inflammation.^6^^,^^7^ Theories attribute this cross-talk to innate lymphoid cells (ILCs), which can be induced to migrate out of the gut to distant tissues including the lung, and toll-like receptor (TLR) signaling,^6^^,^^8–10^ all of which may contribute to causing inflammation within the lungs. Additionally, medication-related pulmonary toxicity is an important consideration in IBD patients, and may be difficult to distinguish from IBD-associated pulmonary involvement, as certain biologic and non-biologic medications have been associated with pulmonary toxicity and ILD.^11–13^

Overall, respiratory involvement in IBD is frequently overlooked, with an insidious onset and having a multifactorial etiology.^11^^,^^14^ Recognizing the prevalence of ILD in IBD patients and understanding the many risk factors at play may help facilitate screening, allowing earlier diagnosis and management, and result in improved long-term patient outcomes.^15^ The aim of this study is to determine the association of ILD with CD and UC, and to analyze the underlying contributory factors along with the impact of ILD on healthcare utilization and patient outcomes.

## Methods

A retrospective chart review was conducted using validated medical record numbers of patients. Data were exported from the electronic health records of these patients, who were seen at one of two major tertiary care centers in the southeastern United States from January 1, 2012, to December 31, 2022. Patients were identified using International Classification of Diseases codes (ICD-9 and ICD-10) that corresponded to UC and CD. Patients were excluded if they were not seen in the health system’s gastroenterology clinics at least once or if their demographic information was incomplete. Data specifically included information on patient demographics, healthcare utilization patterns, and coexisting diagnoses of ILD.

Information was extracted from the electronic health record charts of patients and compiled, first identifying the total number of patients with IBD (both UC and CD), who fit the above criteria. A total of 8865 IBD patients met the inclusion criteria, and 408 of those had comorbid ILD.

ILD presents in many different forms and disease patterns, ranging from idiopathic pulmonary fibrosis (IPF) to sarcoidosis to being secondary manifestations of connective tissue disorders. As a result, using the standardized codes and groupings of ICD-9 and ICD-10 codes corresponding to ILD, patients from the following major categories were included (*see Appendix*):

### ICD-9 codes

515: Post-inflammatory pulmonary fibrosis.

516: Other alveolar and parietoalveolar pneumonopathy.

### ICD-10 codes

J84: Other interstitial pulmonary diseases.

IBD patients were included in the IBD-ILD group if they had at least one ICD-9 or ICD-10 code (as detailed above) that correlated with ILD. Additionally, 100 randomly selected patient charts were manually reviewed and compared with the exported data set to validate that the exported information was accurate in comparison to patient charts.

Population characteristics, demographics, healthcare utilization (emergency department (ED) visits and hospitalizations), prescription data, and race were compared amongst the IBD-only and IBD-ILD groups. Statistical analysis was performed with *t*-tests and χ^2^ tests. Following univariate analysis, a multivariate logistic model was created to control for potential confounding variables. Results with a *P*-value <.05 were considered to be statistically significant.

### Ethical considerations

Institutional review board (IRB) approval was obtained for this retrospective study.

## Results

A total of 8865 IBD patients were included in our study, of which 5352 had a diagnosis of CD and 3513 had a diagnosis of UC. 408 of these patients met the criteria for concomitant IBD and ILD, of which 196 patients had UC and ILD, and 212 had CD and ILD.

We initially examined our entire IBD patient population (*n* = 8865). Among IBD patients as a whole, UC patients were significantly older (*P* < .001), averaged fewer gastroenterology clinic visits (*P* < .001), averaged more hospitalizations (*P* < .001), had a lower proportion of patients with at least one IBD-related surgery (*P* < .001), had a higher proportion of patients who were prescribed mesalamine (*P* < .001), a lower proportion of patients who were prescribed methotrexate (*P* < .001), a lower proportion of patients who were prescribed azathioprine (*P* < .001), and had a lower proportion of patients who were prescribed biologic medications (*P* < .001). All other demographic and healthcare utilization variables lacked any statistically significant differences, as seen in Table 1.

**Table 1:** Stratification of the entire IBD population (*n* = 8865) with various demographic and healthcare utilization factors.

	CD (*n* = 5352)	UC (*n* = 3513)	*P*-value
**Number of males, *N* (%)**	2203 (41.2%)	1448 (41.2%)	.958
**Average age in years (mean ± SD)**	49.7 ± 19.1	52.4 ± 19.4	<.001*
**Average number of clinic visits (mean ± SD)**	13.2 ± 17.2	10.4 ± 12.0	<.001*
**Average number of ED visits (mean ± SD)**	2.7 ± 7.2	2.8 ± 9.0	.563
**Average number of total hospitalizations (mean ± SD)**	2.2 ± 4.8	2.6 ± 5.9	<.001*
**Number of patients with at least one IBD surgery, *N* (%)**	629 (11.8%)	296 (8.4%)	<.001*
**Average number of outpatient steroid prescriptions (mean ± SD)**	1.9 ± 4.2	1.8 ± 4.2	.272
**Number of patients prescribed mesalamine, *N* (%)**	1345 (25.1%)	1822 (51.9%)	<.001*
**Number of patients prescribed methotrexate, *N* (%)**	747 (14.0%)	239 (6.8%)	<.001*
**Number of patients prescribed azathioprine, *N* (%)**	905 (16.9%)	413 (11.8%)	<.001*
**Number of patients prescribed biologic medications, *N* (%)**	1408 (26.3%)	588 (16.7%)	<.001*

*n*, number of patients included in the entire group; *N*, number of patients included in the analyzed subgroup category.

Abbreviations: CD, Crohn’s disease; ED, emergency department; IBD, inflammatory bowel disease; UC, ulcerative colitis.

* statistical significance

When analyzing our subset of 408 IBD-ILD patients, there was a significantly higher prevalence of comorbid ILD among patients with UC compared with CD (5.6% vs 4.0%, respectively; *P* < .001). There were no statistically significant differences in age, sex, ED visits, hospitalizations, average number of corticosteroid prescriptions, or proportion of patients who were prescribed biologic medications, between the UC-ILD and CD-ILD subgroups. When examining individual non-biologic medications, however, UC-ILD patients had a higher proportion of patients prescribed mesalamine (*P* < .001), and a lower proportion of patients prescribed methotrexate (*P* < .001) (Table 2).

**Table 2:** Patients with diagnoses of both IBD and ILD (*n* = 408) with at least one outpatient gastroenterology clinic visit, stratified by type of IBD and various demographic and healthcare utilization factors.

	CD-ILD (*n* = 212)	UC-ILD (*n* = 196)	*P*-value
**Prevalence of concomitant ILD among IBD patients in database (%)**	4.0%	5.6%	<.001*
**Number of males, *N* (%)**	87 (41.0%)	83 (42.3%)	.788
**Average age in years (mean ± SD)**	63.3 ± 16.2	63.7 ± 15.8	.801
**Average number of ED visits (mean ± SD)**	6.6 ± 14.1	7.1 ± 15.6	.734
**Average number of total hospitalizations (mean ± SD)**	8.4 ± 9.3	9.4 ± 11.3	.328
**Number of patients with any corticosteroid use, *N* (%)**	157 (74.1%)	139 (70.9%)	.478
**Average number of outpatient steroid prescriptions (mean ± SD)**	5.0 ± 8.9	6.0 ± 10.6	.301
**Number of patients prescribed mesalamine, *N* (%)**	45 (21.2%)	65 (33.2%)	<.001*
**Number of patients prescribed methotrexate, *N* (%)**	26 (12.3%)	9 (4.6%)	<.001*
**Number of patients prescribed azathioprine, *N* (%)**	26 (12.3%)	29 (14.8%)	.454
**Number of patients prescribed biologic medications, *N* (%)**	35 (16.5%)	22 (11.2%)	.124

*n*, number of patients included in the entire group; *N*, number of patients included in the analyzed subgroup category.

Abbreviations: CD, Crohn’s disease; ED, emergency department; IBD, inflammatory bowel disease; ILD, interstitial lung disease; UC, ulcerative colitis.

* statistical significance

When the 408 patients with IBD-ILD were stratified by race, 292 patients had self-identified as White and 107 had self-identified as minorities. Nine patients were of unknown race or chose not to identify as any one race (Table 3). There was a significant difference in prevalence between White patients and minority patients with IBD and any form of ILD (*P* < .001).

**Table 3:** Proportion of patients with comorbid IBD and ILD (*n* = 408) among total IBD patients (*n* = 8865), stratified by race.

	Concomitant IBD-ILD (*n* = 408)	*P*-value
**White patients (*n* = 6725), *N* (%)**	292 (4.3%)	
**Minority patients (*n* = 1594), *N* (%)**	107 (6.7%)	<.001*
**Unidentified patients (*n* = 546), *N* (%)**	9 (1.6%)	

*n*, number of patients included in the entire group; *N*, number of patients included in the analyzed subgroup category.

Abbreviations: IBD, inflammatory bowel disease; ILD, interstitial lung disease.

* statistical significance

Finally, demographics, healthcare utilization, and prescription data were compared among patients with IBD-only versus patients with comorbid IBD-ILD. There were no statistically significant differences in sex or in prescription of methotrexate or azathioprine noted. However, regarding all other factors examined, there were statistically significant differences seen (Table 4). Patients with IBD-ILD were significantly older (*P* < .001), had a higher proportion of patients with an ED visit (*P* < .001), had a higher proportion of patients who had been hospitalized (*P* < .001), had a higher proportion of patients with an IBD-related surgery (*P* < .001), and had a higher proportion of patients with steroid use (*P* < .001). Of note, the proportion of patients with IBD-ILD who were prescribed mesalamine, and those who were prescribed biologic medications was significantly lower compared to those with IBD-only (*P* < .001).

**Table 4:** Demographics, healthcare utilization, and prescription data among patients with IBD only, compared to patients with comorbid IBD-ILD.

	IBD only (*n* = 8457)	IBD-ILD (*n* = 408)	*P*-value
**Number of males, *N* (%)**	3481 (41.2%)	170 (41.7%)	.839
**Average age in years (mean ± SD)**	50.2 ± 19.2	63.5 ± 16.0	<.001*
**Number of patients with at least one ED visit, *N* (%)**	3932 (46.5%)	293 (71.8%)	<.001*
**Average number of ED visits (mean ± SD)**	2.5 ± 7.4	6.8 ± 14.8	<.001*
**Number of patients with at least one hospitalization, *N* (%)**	4121 (48.7%)	368 (90.2%)	<.001*
**Average number of hospitalizations (mean ± SD)**	2.1 ± 4.7	8.9 ± 10.3	<.001*
**Number of patients with at least one IBD-related surgery, *N* (%)**	859 (10.2%)	66 (16.2%)	<.001*
**Number of patients with any corticosteroid use, *N* (%)**	3829 (45.3%)	296 (72.5%)	<.001*
**Number of patients prescribed mesalamine, *N* (%)**	3057 (36.1%)	110 (27.0%)	<.001*
**Number of patients prescribed methotrexate, *N* (%)**	951 (11.2%)	35 (8.6%)	.094
**Number of patients prescribed azathioprine, *N* (%)**	1263 (14.9%)	55 (13.5%)	.420
**Number of patients prescribed biologic medications, *N* (%)**	1939 (22.9%)	57 (14.0%)	<.001*

*n*, number of patients included in the entire group; *N*, number of patients included in the analyzed subgroup category.

Abbreviations: ED, emergency department; IBD, inflammatory bowel disease; ILD, interstitial lung disease.

* statistical significance

A multivariate model was constructed as detailed in the Methods section in order to control for potential confounding variables and to adjust for covariates (age [in 10-year intervals], sex, number of ED visits, number of hospitalizations, history of IBD-related surgeries, prescription of biologic medications, and total grams of corticosteroids prescribed). In multivariate logistic regression analysis, age [in 10-year intervals] odds ratio [OR] 1.45, 95% confidence Interval [CI], 1.36-1.54; *P* < .001), number of hospitalizations (OR 1.10, 95% CI, 1.09-1.12; *P* < .001), and grams of corticosteroids prescribed (OR 1.01, 95% CI, 1.004-1.010; *P* < .001) were independently associated with the diagnosis of comorbid ILD. The prescription of biologic medications did not reach the pre-set level of statistical significance for our study (OR 0.75, 95% CI, 0.546-1.020; *P* = .067). Sex, number of ED visits, and history of IBD-related surgeries were not significantly associated with diagnosis of comorbid ILD.

## Discussion

The relationship between IBD and ILD remains underexplored, with relatively limited data in literature. A recent study using Mendelian randomization was able to identify a causal relationship between IBD and ILD.^16^ In a recently conducted nationwide population-based cohort study in Sweden consisting of over 85 000 patients, those with IBD had a significantly higher relative risk of ILD.^15^ Additionally, they showed that women with IBD had a higher relative risk of being diagnosed with ILD than men, with no other significant differences in sub-group analyses of healthcare utilization.^15^ Another retrospective analysis identified the increased prevalence of various pulmonary diseases in IBD, but with no specific mention of ILD.^13^ One multicenter retrospective study with a small sample size of 31 ILD patients found that the majority had UC.^17^ Finally, another U.S. population-based retrospective epidemiological study observed an increase in mortality rates over time due to ILD among IBD patients.^11^

In our retrospective analytical study, we found that ILD is significantly more associated with UC in comparison with CD. Retrospective analysis demonstrated that 5.6% of patients had concomitant diagnoses of UC and ILD, compared to 4.0% with CD and ILD (*P* < .001). Our findings are in line with the current literature where ILD as an extraintestinal manifestation of IBD has been more strongly associated with UC.^15^^,^^17^ Kochar et al. demonstrated a higher incidence rate of ILD in UC patients compared to CD patients, and Eliadou et al. demonstrated that most patients out of their entire subset of IBD-ILD patients had UC, however, with a heavy prevalence of drug-related ILD in these patients.^15^^,^^17^

When examining specific contributory factors, no statistically significant differences in demographics, healthcare utilization, or the prescription of biologic medications were seen among UC-ILD patients and CD-ILD patients. However, there was a significantly greater prevalence of UC-ILD patients who were prescribed mesalamine and a significantly lower prevalence of UC-ILD patients who were prescribed methotrexate. This is expected given that mesalamine is a standard first-line therapy for patients with mild or moderate UC, whereas its effectiveness in CD is limited. These results emphasize the need for additional studies to further stratify the differential increased proportion of ILD in UC patients compared to CD patients.

There was a significantly increased proportion of ILD in minority IBD patients, seen in our study (Table 3). Within the literature, there is limited and conflicting information regarding differences in EIMs across racial and ethnic groups. Some studies point to no major differences in EIMs among races and ethnicities.^18^^,^^19^ When examining the EIM of ILD specifically, only one other study describing an increased prevalence of ILD in African American IBD patients compared to Caucasian IBD patients was identified.^20^ Racial and ethnic stratification of ILD in IBD remains a significant gap in the epidemiologic literature of ILD in IBD.

As it pertains to gender stratification, within the literature, when examining specific types of ILD, the idiopathic pulmonary fibrosis (IPF) form of ILD is seen more commonly in males, whereas connective tissue disease–related ILDs are seen more commonly in females.^21^ In the context of IBD, given that our study evaluated ILD as an entire entity and disease category, rather than examining the different subtypes of ILD, we did not observe any gender predominance between CD-ILD and UC-ILD (Table 2), and between the IBD-only and IBD-ILD populations (Table 4). Future studies focusing on the prevalence of specific subtypes of ILD with IBD would provide interesting insights as to whether this gender stratification seen in ILD subtypes also exists in concomitant IBD-ILD diagnosed patients.

Similarly, the average age of our population of CD-ILD and UC-ILD patients was nearly identical, implying that the differential prevalence of CD-ILD and UC-ILD is not associated with age. In the literature, UC has been seen more commonly in the elderly with higher incidence rates in individuals over the age of 60.^15^^,^^22^

Pertaining to healthcare utilization, regarding average number of ED visits and average number of hospitalizations, there was no observed significant difference between CD-ILD and UC-ILD patients. Results did not indicate whether UC-ILD or CD-ILD places a greater financial burden on the healthcare system. Additionally, there was no significant difference in medication usage with corticosteroid use or biologic medication prescriptions. Available research has demonstrated that compared to IBD alone, comorbid IBD-ILD has a greater healthcare burden, as would be expected.^17^ Additionally, due to the more severe outcomes seen in UC-ILD, the burden is generally higher in these patients, contrary to our study’s findings.^17^

Comparing the IBD-ILD subset of patients with patients with IBD only also revealed interesting findings. As discussed previously, there were no statistically significant differences in sex. However, patients with comorbid IBD-ILD were significantly older, as would be expected, as ILD is a slow progressive disease with a later peak onset age between 50 and 60 years of age. Additionally, comorbid IBD-ILD patients had significantly greater healthcare utilization patterns, as ILD places a greater healthcare burden on patients, requiring increased acuity of care, in line with the literature.^17^ Regarding non-biologic medications, there was a significantly lower prevalence of IBD-ILD patients who were prescribed mesalamine compared to IBD only patients, with no statistically significant differences noted with the use of other non-biologic medications studied, including methotrexate and azathioprine. This suggests that methotrexate and azathioprine were not disproportionately observed among patients with comorbid IBD-ILD and that the presence of ILD was not associated with substantial changes in methotrexate and azathioprine prescription patterns within this cohort. The decreased prevalence of mesalamine prescription among patients with IBD-ILD compared to IBD only patients presents an interesting finding, with several possible explanations. IBD-ILD is associated with greater disease severity, and thus patients may be more likely to have received more intense biologic therapies instead of mesalamine. Additionally, the decreased usage of mesalamine may also suggest concerns with mesalamine-induced lung toxicity either through potentially contributing to the development of ILD in IBD patients, or general avoidance of the medication by prescribers due to known concerns of its pulmonary toxicity. Finally, there was a significantly lower prevalence of comorbid IBD-ILD patients who had received biologic therapy compared to IBD only patients. Some possible explanations exist, as biologic medications could help protect against ILD or biologics could decrease systemic inflammation, making it difficult to identify and diagnose ILD. Further research must be conducted on the role of biologics and non-biologics in the development or prevention of ILD, on the inflammatory cascade seen in IBD-ILD, and whether these medications may provide additional benefits in addition to their standard of care in the treatment of IBD.

These interesting findings were further investigated through multivariate logistical analysis to control for confounding variables. In doing so, we were able to identify that age significantly increases the chance of an ILD diagnosis in IBD patients. Additionally, the number of ED visits, sex of patients, and total number of IBD-related surgeries were not significantly associated with the diagnosis of comorbid ILD.

As discussed above, and seen in literature, the role of biologics in ILD presents a mixed picture, as some biologics provide a therapeutic effect in treating ILD, with stabilization of lung function, while others often pose the risk of worsening ILD. Young patients are less likely to be diagnosed with ILD, and it is well established that younger IBD patients are more likely to require biologic medications. This may explain why biologic use was associated with a significantly decreased prevalence of comorbid IBD-ILD in univariate analysis but did not reach the level of statistical significance in multivariate modeling (which included age as a covariate). Alternatively, biologic use itself could provide protective effects against systemic and lung inflammation, reducing the odds of being diagnosed with ILD. Studying the intricate role of biologic medications on the development or prevention of ILD may provide breakthroughs in the understanding of the long-term care and treatment of IBD-ILD as a whole.

Inherent limitations in our study exist, due to its retrospective nature, which can often lead to incomplete data, and make it difficult to establish causality. While a strict process of using ICD codes to identify concomitant IBD-ILD patients was used, patients who may have had incomplete documentation of diagnoses despite having these diseases would be excluded from the data set. Additionally, while retrospective analysis using ICD codes allows for large-scale, data-driven models, the obvious limitation is that the diagnoses are not and sometimes cannot be confirmed.

Our study was conducted across two large tertiary care centers from 2012 to 2022. As such, some patients were diagnosed at outside facilities and then referred to our institution, were diagnosed prior to adopting electronic health records in 2012, or were impacted by the coronavirus disease pandemic (COVID-19) from 2020 onward, where patients with COVID-19-associated disease may have been mistakenly labeled with an ICD code for ILD. Overall, 324 of 408 (79.4%) patients with comorbid IBD-ILD underwent either pulmonary function testing (PFTs) or chest computed tomography (CT) imaging at one of our facilities, providing additional support for ILD diagnostic ascertainment and accuracy. Nonetheless, these limitations should be considered when interpreting certain findings in our study.

Finally, certain non-biologic IBD medications can cause pulmonary toxicity and confound the observed prevalence, though the data for methotrexate and azathioprine were not significantly disproportionate in patients with IBD-ILD compared to patients with IBD only. Sulfasalazine can also cause pulmonary toxicity, however, we did not have data available for sulfasalazine in this study. Furthermore, because the temporal relationship between medication usage and ILD diagnosis could not be fully established in this retrospective study, this remains a potential source of residual confounding, which should be considered when interpreting the relationship between IBD therapy and ILD.

Future research focusing on the relationship between IBD and ILD is necessary to corroborate our findings and to further determine the factors contributing to the variation among UC-ILD and CD-ILD patients and IBD-ILD patients compared to IBD only patients. We were only able to identify one study, by Kochar et al., conducted in Sweden, demonstrating an increased risk of developing ILD in IBD patients, with a higher incidence rate of ILD seen in UC compared to CD.^15^ Due to the increased healthcare burden that IBD-ILD poses on patients, early diagnosis and screening of ILD should be considered in all IBD patients, and perhaps in resource-limited settings, should focus on UC patients. Finally, investigation into the pathophysiology behind the relationship between IBD and ILD could also provide clinicians new information regarding modes of screening, diagnosis, and treatment, particularly with the role of biologic medications in ILD, as an EIM of IBD.

To our knowledge, this is the largest cohort of IBD-ILD patients ever analyzed in the United States. Although future prospective studies may be useful, it would prove extremely difficult to amass such a cohort of IBD-ILD patients in a real-world prospective study, as evidenced by the fact that no such study exists to date.

## Conclusion

Our comprehensive review of ILD in IBD patients showed that ILD is more associated with UC compared with CD, without any statistically significant differences in underlying demographic and healthcare utilization factors, apart from the prescription of certain non-biologic medications. Minority patients had a statistically significantly increased prevalence of comorbid IBD and ILD compared to White patients, and comorbid IBD-ILD patients had significantly greater healthcare utilization patterns. Finally, the role of biologic and non-biologic medications presents a mixed picture as it pertains to the prevalence of comorbid IBD-ILD. Our results emphasize the importance of further research on the relationship between IBD and ILD, with a focus on underlying demographic factors and the role of biologic and non-biologic medications.

## Supplementary Material

otag085_Supplementary_Data

## Data Availability

Data are not publicly available.

## References

[1] Levine JS , Burakoff R. Extraintestinal manifestations of inflammatory bowel disease. Gastroenterol Hepatol (N Y). 2011;7:235-241. 21857821 PMC3127025

[2] Camus P , Piard F, Ashcroft T, et al The lung in inflammatory bowel disease. Medicine (Baltimore). 1993;72:151-183. 8502168

[3] Maher TM. Interstitial lung disease: a review. JAMA. 2024;331:1655-1665. 10.1001/jama.2024.3669 38648021

[4] Wijsenbeek M , Suzuki A, Maher TM. Interstitial lung diseases. Lancet. 2022;400:769-786. 10.1016/S0140-6736(22)01052-2 35964592

[5] Althobiani MA , Russell AM, Jacob J, et al Interstitial lung disease: a review of classification, etiology, epidemiology, clinical diagnosis, pharmacological and non-pharmacological treatment. Front Med (Lausanne). 2024;11:1296890. 10.3389/fmed.2024.1296890 38698783 PMC11063378

[6] Ghosh S , Deshwal H, Haraf R, et al Pulmonary manifestations of inflammatory bowel disease and treatment strategies. CHEST Pulm. 2023;1:100018. 10.1016/j.chpulm.2023.100018 42548539 PMC13418686

[7] Metwali A , Thorne PS, Ince MN, et al Recirculating immunocompetent cells in colitic mice intensify their lung response to bacterial endotoxin. Dig Dis Sci. 2018;63:2930-2939. 10.1007/s10620-018-5196-z 30022451 PMC6182434

[8] Mjösberg J , Rao A. Lung inflammation originating in the gut. Science. 2018;359:36-37. 10.1126/science.aar4301 29302003

[9] Ichinohe T , Pang IK, Kumamoto Y, et al Microbiota regulates immune defense against respiratory tract influenza a virus infection. Proc Natl Acad Sci U S A. 2011;108:5354-5359. 10.1073/pnas.1019378108 21402903 PMC3069176

[10] Zhou X , Liao Y. Gut-lung crosstalk in sepsis-induced acute lung injury. Front Microbiol. 2021;12:779620. 10.3389/fmicb.2021.779620 35003009 PMC8733643

[11] Vaezi A , Ashby T, Schweitzer M, et al Interstitial lung disease as an emerging contributor to mortality in patients with inflammatory bowel disease: a population-based epidemiological study. Clin Transl Gastroenterol. 2024;15:e1. 10.14309/ctg.0000000000000720 PMC11421727 38822801

[12] Storch I , Sachar D, Katz S. Pulmonary manifestations of inflammatory bowel disease. Inflamm Bowel Dis. 2003;9:104-115. 10.1097/00054725-200303000-00004 12769444

[13] Pemmasani G , Loftus EV, Tremaine WJ. Prevalence of pulmonary diseases in association with inflammatory bowel disease. Dig Dis Sci. 2022;67:5187-5194. 10.1007/s10620-022-07385-z 35142913

[14] Karadag F , Ozhan MH, Akçiçek E, et al Is it possible to detect ulcerative colitis-related respiratory syndrome early? Respirology. 2001;6:341-346. 10.1046/j.1440-1843.2001.00347.x 11844126

[15] Kochar B , Sun J, Olén O, et al Inflammatory bowel disease patients are at increased risk for interstitial lung diseases. Am J Gastroenterol. 2026;121:1455-1461. 10.14309/ajg.0000000000003699 40758133

[16] Luo Q , Zhou P, Chang S, et al The gut-lung axis: Mendelian randomization identifies a causal association between inflammatory bowel disease and interstitial lung disease. Heart Lung. 2023;61:120-126. 10.1016/j.hrtlng.2023.05.016 37247539

[17] Eliadou E , Moleiro J, Ribaldone DG, et al Ecco Confer Committee. Interstitial and granulomatous lung disease in inflammatory bowel disease patients. J Crohns Colitis. 2020;14:480-489. 10.1093/ecco-jcc/jjz165 31602473

[18] Afzali A , Cross RK. Racial and ethnic minorities with inflammatory bowel disease in the United States: a systematic review of disease characteristics and differences. Inflamm Bowel Dis. 2016;22:2023-2040. 10.1097/MIB.0000000000000835 27379446

[19] Mahid SS , Mulhall AM, Gholson RD, et al Inflammatory bowel disease and african americans: a systematic review. Inflamm Bowel Dis. 2008;14:960-967. 10.1002/ibd.20389 18266229

[20] Alchirazi KA , Eltelbany A, Mohammed A, et al S933 racial disparities in extraintestinal manifestations in patients with inflammatory bowel disease. Am J Gastroenterol. 2022;117:e675-e676. 10.14309/01.ajg.0000860372.72767.3c

[21] Ozaki M , Glasgow A, Oglesby IK, et al Sexual dimorphism in interstitial lung disease. Biomedicines. 2022;10:3030. 10.3390/biomedicines10123030 36551792 PMC9775147

[22] Arnott I , Rogler G, Halfvarson J. The management of inflammatory bowel disease in elderly: Current evidence and future perspectives. Inflamm Intest Dis. 2018;2:189-199. 10.1159/000490053 30221146 PMC6135226

