# Supplementary material for: Confirming the association of ulcerative colitis with interstitial lung disease: a retrospective analysis of comorbid disease, demographics, and healthcare utilization
Source: Crohns Colitis 360. 2026 Jul 24;8(3):otag085. doi: 10.1093/crocol/otag085 (PMC13449601; doi:10.1093/crocol/otag085)
Supplement: Supplementary file 1 [file otag085_supplementary_data.docx]

**Appendix:**

*ICD-9 Codes included:*

515 Post inflammatory pulmonary fibrosis

516 Other alveolar and parietoalveolar pneumonopathy

516.0 Pulmonary alveolar proteinosis

516.1 Idiopathic pulmonary hemosiderosis

516.2 Pulmonary alveolar microlithiasis

516.3 Idiopathic interstitial pneumonia

516.30 Idiopathic interstitial pneumonia, not otherwise specified

516.31 Idiopathic pulmonary fibrosis

516.32 Idiopathic non-specific interstitial pneumonitis

516.33 Acute interstitial pneumonitis

516.34 Respiratory bronchiolitis interstitial lung disease

516.35 Idiopathic lymphoid interstitial pneumonia

516.36 Cryptogenic organizing pneumonia

516.37 Desquamative interstitial pneumonia

516.4 Lymphangioleiomyomatosis

516.5 Adult pulmonary Langerhans cell histiocytosis

516.6 Interstitial lung diseases of childhood

516.69 Other interstitial lung diseases of childhood

516.8 Other specified alveolar and parietoalveolar pneumonopathies

516.9 Unspecified alveolar and parietoalveolar pneumonopathy

*ICD-10 Codes included:*

J84 Other interstitial pulmonary diseases

J84.0 Alveolar and parieto-alveolar conditions

 	J84.01 Alveolar proteinosis

 	J84.03 Idiopathic pulmonary hemosiderosis

 	J84.09 Other alveolar and parieto-alveolar conditions

 J84.1 Other interstitial pulmonary diseases with fibrosis

 	J84.10 Pulmonary fibrosis, unspecified

 	J84.11 Idiopathic interstitial pneumonia

 	J84.111 Idiopathic interstitial pneumonia, not otherwise specified

 	J84.112 Idiopathic pulmonary fibrosis

 	J84.113 Idiopathic non-specific interstitial pneumonitis

J84.114 Acute interstitial pneumonitis

J84.115 Respiratory bronchiolitis interstitial lung disease

J84.117 Desquamative interstitial pneumonia

J84.17 Other interstitial pulmonary diseases with fibrosis in diseases classified elsewhere

J84.170 Interstitial lung disease with progressive fibrotic phenotype in diseases classified elsewhere

J84.178 Other interstitial pulmonary diseases with fibrosis in diseases classified elsewhere

 J84.2 Lymphoid interstitial pneumonia

 J84.8 Other specified interstitial pulmonary diseases

 J84.81 Lymphangioleiomyomatosis

 J84.82 Adult pulmonary Langerhans cell histiocytosis

 	J84.89 Other specified interstitial pulmonary diseases

 J84.9 Interstitial pulmonary disease, unspecified
